# Visualizing the activity of *Escherichia coli* divergent promoters and probing their dependence on superhelical density using dual-colour fluorescent reporter vector

**DOI:** 10.1038/srep11449

**Published:** 2015-06-17

**Authors:** Irina S. Masulis, Zaira Sh. Babaeva, Sergey V. Chernyshov, Olga N. Ozoline

**Affiliations:** 1 Laboratory of functional genomics and cellular stress, Institute of Cell Biophysics, Russian Academy of Sciences, Pushchino, Moscow region, 142290, Russian Federation; 2 Group of molecular biotechnology, Branch of Shemyakin & Ovchinnikov Institute of Bioorganic Chemistry, Russian Academy of Sciences, Pushchino, Moscow region, 142290, Russian Federation

## Abstract

Mosaic pattern of transcription in alternating directions is a common feature of prokaryotic and eukaryotic genomes which rationality and origin remain enigmatic. In *Escherichia coli* approximately 25% of genes comprise pairs of topologically linked divergently transcribed units. Given that transcriptional complex formation at each promoter in the pair induces topological changes and is itself sensitive to DNA structural perturbations, study of the functional anatomy in such areas requires special approaches. Here we suggested the dual-colour promoter probe vector which may become an ideal tool for divergent transcription profiling. The vector was used to characterize the specific genomic region nearby *app*Y with multiple bidirectional promoters predicted *in silico*. Only three promoters of this region were shown to be engaged in the transcription initiation resulting in the expression of reporter genes. RNA product transcribed in antisense direction is suggested as a novel RNA. Nalidixin-induced topological modulation differentially affected transcription in sense and antisense directions thus exemplifying anticooperative mode in the response to topological alterations.

It is already well established that global and local variations in superhelical density can affect the rate and efficiency of gene expression in bacteria^1^,^2^. Such alterations mediate cellular response to environmental changes^3^ and are controlled by many physiological factors. Besides gyrases and topoisomerases I and IV, keeping certain level of superhelicity depending on intracellular stimuli^4^, local topological transitions can also be caused by proteins unwinding or bending DNA upon interaction or impeding its equilibrium relaxation. Divergently oriented neighbouring genes, separated by a distance from dozen to hundreds of base pairs exemplify a special type of position-dependent topology-mediated regulation. Previously it was considered as an evolutionary selected way to balance required expression levels via optimizing gene order^5^. However, up to now this type of regulation remains the most obscure issue in modern transcriptomics, because simultaneously transcribed closely located genes change the local genetic environment in the common regulatory region making their transcriptional output hardly predictable.

Data regarding the effects of DNA topology on transcription were accumulated since early 80^th^ 6 and classical model of transcription-induced formation of domains with positive and negative supercoiling ahead and behind of transcribing RNA polymerase^7^ is now generally accepted. From this view point divergent promoters initiating transcription in opposite directions cooperate in each other functioning. In total, 602 pairs of oppositely oriented genes are distributed along the entire chromosome of *E.coli* (http://regulondb.ccg.unam.mx/)^8^. Each pair of such genes may be considered as a particularly sensitive matter, responding to the entire structural background and also to the functional state of each other. Hence, about 25% of total number of *E.coli* genes could be subjected to complex regulation mediated by superposition of global and local changes in superhelicity defined as supercoiling-dependent transcriptional coupling^9^.

The role of divergent transcription, its topological consequences and dependence on overall DNA structure seems to become more generalized if the data of computational predictions^10^ and throughoutput RNA sequencing^11^ were accounted. Both these data indicated high abundance of closely spaced bidirectional promoters capable to produce known RNAs or oligonucleotides with unknown functions. Particular importance in this regard may have areas with unusually high density of potential promoters (“promoter islands”). Seventy-eight such “islands” were recently found^10^ and partly characterized^12^,^13^ in the genome of *E.coli*. Topological drift in these regions in response to multiple functional interactions with proteins of transcription machinery is expected with the highest probability.

For eukaryotes the role of divergent promoters in coordinated gene expression and transcription noise suppression was extensively discussed over the last decade^14^,^15^. However, for the bacterial genomes the importance of this type of functional connections is not yet fully understood, even though the molecular mechanisms operating in oppositely transcribed regions in some cases have been studied in details. Thus, Rhee and co-authors^16^ have found that negative supercoiling, generated behind strong promoter ilvY, activated its back-to-back promoter ilvC and it was demonstrated that supercoiling-dependent promoter leu-500 of *Salmonella typhimurium* activated in topA mutant remained functional if this mutation was suppressed^17^. An absence of expected decrease in promoter activity was explained by the presence of another functional promoter on the opposite strand.

To study the relative activity of oppositely oriented promoters of *E.coli* we constructed a promoterless reporter vector divergently expressing genes of two colour proteins EGFP (green) and mCherry (red). In order to address how changes in DNA superhelical density may affect divergent transcription, we treated cells with low concentrations of gyrase inhibitor nalidixic acid and monitored activities of two overlapping promoters for *fep*A and *fes* genes and promoters of *app*Y-associated “promoter island”. We found that both promoter regions responded to the decrease in negative supercoiling and in both cases changes in the relative activity of divergent promoters were registered, but the scale of effect was greater in the region with multiple promoter-like signals, which assumes its higher topological flexibility. Given that “promoter islands” are more bent and overtwisted than other genomic regions tested so far^12^, while the dynamic behavior of DNA is strongly dependent on the equilibrium sequence-dependent trajectory^18^, promoter-rich regions may be considered as a natural sensors of global structural perturbations in genomic DNA.

## Results

### Construction of a reporter vector for bidirectional transcription

**Figure 1:**
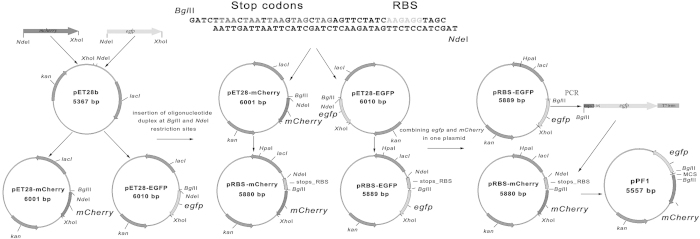
Scheme illustrating successive steps in the construction of pPF1.

Bidirectional promoter probe vectors have been proposed as a useful tool for divergent transcription studying about twenty years ago. The first systems exploited genes of different enzymes with easily detectable activity, such as β-galactosidase, alkaline phosphatase or bacterial luciferase^19^,^20^. The widespread use of fluorescent proteins has created the basis for a new generation of reporter systems. They have advantage of simultaneous measurements of both reporter genes expression using identical rather then enzyme-specific modes of registration. Here we introduce the dual-colour vector derived from the pET-28b plasmid and supplemented with two genes encoding fluorescent proteins EGFP and mCherry. Integrated genes were PCR-amplified from plasmids pEGFP-N3 and pmCherry-C1 (Fig. 1) using one and the same pair of primers (GFP-NdeI and GFP-Xho) since 22 bp in their C-terminal parts, and 21 bp of N-termini are identical. As a result, two intermediate plasmids - pET28-EGFP and pET28-mCherry were obtained (Fig. 1).

A ribosome binding site with a context AAGAGG preceded by a series of translational terminators for all three possible ORFs was integrated between BglII and NdeI restriction sites in each plasmid. The insert was prepared by annealing of two oligonucleotides, BglII-stops-RBS and NdeI-RBS-stops (Table S1), resulting in the formation of duplex with sticky ends adopted for the ligation into the plasmid treated by BglII and NdeI (Fig. 1). Plasmids, modified by the insertion of RBS and translational terminators were designated as pRBS-EGFP and pRBS-mCherry. They ensured the efficient synthesis of each reporter protein and prevented unauthorized translation from the insert. As a result of these manipulations, T7 promoter, lac operator and His-Taq-coding sequence, not essential for the presumed application, were replaced by the short “stops-RBS” oligonucleotide duplex (Fig. 1). At the next step, *egfp* gene fused with RBS and translational terminators was transferred from the pRBS-EGFP plasmid into the pRBS-mCherry and inserted between BglII and HpaI restriction sites. Inserted fragment was generated with primers “stops-BglII” and “T7term-rev”. Finally BglII site located between two reporter genes was used to integrate “multiple cloning site” with the context given in Supplementary Table S1 (oligonucleotides MCS-forw and MSC-rev). Resulting pPF1 plasmid has perfect mirror symmetry with respect to the cloning site thus setting the same conditions for translation of both reporter proteins.

### Functional validation of the reporter vector using divergent promoters fepA and fes

**Figure 2:**
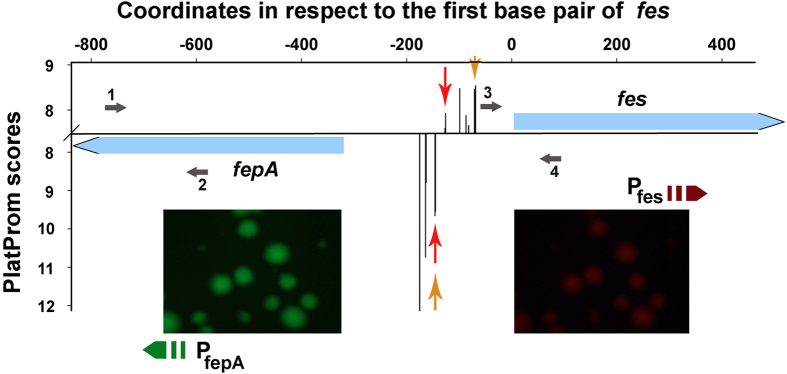
Functional organization of the *fepA/fes* genomic region. Blue arrows show disposition of genes. Black bars show the transcription start points, predicted by the promoter finder PlatProm^10^. Red arrows indicate positions of starts, registered by biochemical approaches^21^ while yellow arrows pointed positions revealed by the 5’-end-specific RNA-seq^11^. Images below exemplify fluorescence of colonies of *E.coli* K12 MG1655 transformed by reporter plasmid carrying the fragment with regulatory region of *fep*A and *fes* integrated between *egfp* and *mCherry*. Positions of primers used for PCR amplification of integrated fragment (primers 1 and 4) and qRT-PCR (primers 1–2 and 3–4) are indicated by small gray arrows.

To test the applicability of the reporter plasmid the fragment containing the known promoters of two divergent genes *fep*A and *fes* (Fig. 2), was incorporated into pPF1 between *egfp* and *mCherry*, and the accumulation of both fluorescent proteins was detected (Fig. 2, images below the plot). The transcription start points of these two well studied promoters^21^ are separated by only 19 bp, i.e. they both should similarly accept topological transitions induced by the functioning at the moment partner. The emission intensities were measured in sonicated cell extracts, corrected per background and the ratio of green and red fluorescence (I_EGFP_/I_mCherry_) was estimated as the most sensitive parameter. Based on these measurements, transcriptional activity of *fep*A promoter, driving expression of *egfp*, appeared to be 5.58 ± 1.66-fold greater than that of *fes* promoter controlling transcription of *mCherry*. This prevalence of fepA over fes promoter is consistent with their relative scores estimated *in silico* (Fig. 2). The same ratio reported by Hunt and co-authors^22^ was much lower (1.3). Promoter activity in that case have been measured using bidirectional vector with genes of β-galactosidase (*lac*Z) and alkaline phosphatase (*pho*A), which intrinsic properties may contribute to registered relative sensitivity. Moreover this distinction may be due to the different experimental conditions used. Thus, we grew cells in standard Luria-Bertani medium, while Hunt and colleagues^22^ either supplemented it with Fe^2+^ ions and sodium citrate or created conditions of iron starvation by the presence of 2,2′-dipyridyl. Furthermore, in our case the expression was measured after 8 h of cultivation to decrease variability and accumulate fluorescent protein, whereas in the cited work it was assayed in early exponential phase. Finally, the promoter-containing regions inserted into reporter vectors were also different. The fragment used in^22^ included the complete sequence of gene *fes* and 5’-part of *fep*A, while we incorporated genomic region containing 20% of *fep*A and about 8% of *fes*.

In order to obtain independent estimate of fepA and fes relative strengths we measured their activities within the native chromosome, using qRT-PCR and highly expressed *rpo*B as a reference gene. Relative amount of *fep*A-mRNA appeared to be 2.5-fold greater than that of *fes*-mRNA (ΔC_t(fepA)_ = 3.78 ± 0.7, n = 7 versus ΔC_t(fes)_ = 5.11 ± 0.74, n = 7), which is intermediate between the estimates discussed above. Realtive promoter activities may therefore depend on experimental conditions and detecting systems used, but all measurements testified a higher activity of fepA over fes promoter.

### Visualizing the relative activity of divergent promoters within the appY-associated “promoter island”

**Figure 3:**
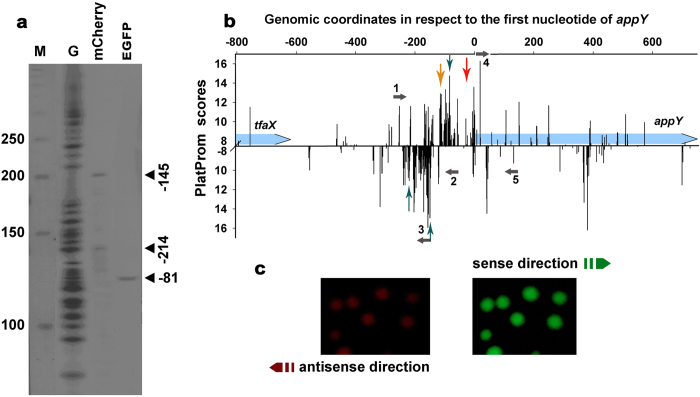
Transcription profiling within the *app*Y-associated “promoter island”. **a:** Products of reverse transcription with ^32^P –labeled oligonucleotide primers specific to *egfp* and *mCherry*. The total fraction of RNAs was isolated from the cells transformed by the reporter vector and used as a template. Positions of cDNA-products are indicated in respect to *app*Y initiation codon. The distances from EGFP-specific and mCherry-specific primers to the insertion site were 101 and 99 bp, respectively. Gel was calibrated by DNA markers (NEB) labeled by [γ-^32^P] in the exchange reaction with T4 polynucleotide kinase and by products of guanine-specific hydrolysis of DNA-fragment ^32^P –labeled at the *mCherry* side. **b:** Functional organization of the *tfaX-appY* genomic region. Red arrow indicates position of start, registered *in vitro*^24^ while yellow arrow points start position registered by the 5’-end-specific RNA-seq^8^. Blue vertical arrows indicate positions of starts, registered in this study. Other designations are as in Fig. 2. c: Images exemplifying fluorescence of colonies of *E.coli* K12 MG1655 transformed by reporter plasmid transcribing two reporter genes from the promoters of appY associated “island”. Inserted DNA fragment was amplified with primers 1 and 2 (gray horizontal arrows in panel **b**), primers 3, 4 and 5 were used for qRT-PCR.

Typical example of “promoter island” is located nearby *app*Y gene. This genomic region is oversaturated by potential transcription start sites corresponding to multiple overlapping promoters (Fig. 3b). Which ones of numerous potential start points are utilized by RNA polymerase in the “island” and how efficiently different promoters of the “island” initiate productive and abortive RNA synthesis may depend on conditions. Transcription within “island” is often blocked by H-NS at the step of short oligonucleotide synthesis^12^,^13^,^23^. These short RNAs are produced from multiple promoters and the excess of such products can mask functional promoter integrated into regulatory networks of the cell.

Two transcription start points for *appY* are currently indicated in the RegulonDB^8^. They are located 25 and 113 bp upstream of *appY* ATG codon. The proximal start was found only *in vitro*^24^, while another one was registered only by the 5’-end specific RNA-seq^8^. The transcription in the opposite direction in this region was not specifically addressed yet. In order to characterize functional and topological features of this promoter-rich area, the first part of the “island” from the position −260 up to position −61 in respect to the start of *appY* (primers 1 and 2 in Fig. 3b) was inserted into pPF1. This fragment covers unpaired thymines, which were identified by potassium permanganate footprinting in the RNA polymerase complex with linear DNA *in vitro*^10^. Such reactivity usually testifies transition of the polymerase-promoter complex into transcriptionally competent open state which includes local DNA melting nearby the transcription start. High level of transcription was detected in both directions (Fig. 3c). Reverse transcription with primers specific to *egfp* (corresponded to the direction of *app*Y) revealed a unique start at position −81 (±2 bp) (Fig. 3a), which fits well to the most probable start point predicted by PlatProm within integrated fragment in position −85 (Fig. 3b, vertical blue arrows). Activity of more distal promoter, also present in the plasmid (yellow arrow in panel b) was not detected. In antisense direction (primers specific to *mCherry*) we registered two or three RNA products, which may be initiated near positions −145, −214 and −225 (Fig. 3a). Two of them (−145 and −225) correspond well to the promoters predicted *in silico* (blue arrows in panel b). Thus, using reverse transcription and reporter vector we detected divergent RNA synthesis within *app*Y-associated promoter “island” and found novel RNA products transcribed oppositely to *app*Y. Being non-overlapping with a potential *app*Y-mRNA these RNAs can not be considered as regulatory molecules for *app*Y expression operating by base pairing at complementary ends. However, in the *tfa*X/*app*Y intergenic region there is enough space for the novel gene which size and functional role remain to be defined.

Thus, according to the data obtained so far transcription of *app*Y may be initiated at positions −113^8^, in the region −81–85 (Fig. 3a) and/or at position 25 bp upstream of ATG codon^24^. Since chromosomal expression of horizontally transferred *appY*^12^ is strongly suppressed by xenogeneic silencer H-NS, all three transcription start sites were registered by means of special approaches: genome-wide RNA-seq^8^, plasmid-based expression (Fig. 3) and transcription from linear DNA^24^. All of them bring some degree of uncertainty and there is a possibility that transcription of *app*Y in chromosomal DNA initiates at some other of multiple promoters predicted in the appY regulatory region (Fig. 3b). Even if the transfer of promoter-rich region into the plasmid context changed the predominant start of transcription initiation, it certainly preserved the disproportion between the huge number of potential start sites and only few experimentally registered transcription initiation points, which remains a mystery of “promoter islands”.

### Topology modulation changed relative activity of divergently oriented promoters

**Figure 4:**
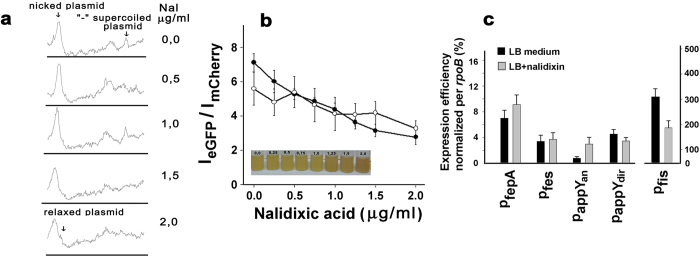
**a:** Dependence of electrophoretic mobility of the reporter plasmid pPF1-*app*Y on nalidixin concentration. The plasmids were isolated from the cells grown in LB for 8 h with indicated concentration of the drug. Fractionation was carried out in 0.8% agarose gel in TAE with 2 μg/ml of chloroquine at 1 V/cm during 20 h. Electrophoretic mobilities are presented as densitograms of corresponding gel lanes. **b:** Dependence of relative emission intensities of EGFP and mCherry on nalidixin concentration. *E.coli* K12 MG1655 cells, transformed by two different reporter plasmids were cultivated during 8 h at given nalidixin concentration. Cells were harvested, suspended in 1xPBS pH 7.4, sonicated and fluorescence was measured in extracts cleared by centrifugation. Wave lengths for excitation/emission were 484/507 nm and 587/610 nm for EGFP and mCherry, respectively. The data are given as a mean values measured in 5 independent biological replicates ± s.e.m. Filled symbols correspond to the promoters of *app*Y-associated “island”, open – to the *fep*A/*fes* regulatory region. Tubes with bacteria cultivated with different concentration of nalidixic acid for 24 h exemplify observed changes in relative promoter activity **c:** The effect of nalidixin (1.5 μg/ml ) on the level of RNAs transcribed from five indicated promoters, estimated by qRT-PCR. Expression efficiency of each gene (E) was calculated by the formula E = 2^−ΔC(t)^ and expressed as a percentage of rpoB-mRNA abundance. Deviation bars show standard error (s.e.m.), number of independent biological replicates for *fep*A, *fes*, *app*Y(dir) were 5; for *fis* n = 6; for *app*Y(an) n = 8.

Nalidixic acid in low concentrations, that had no substantial effect on cell growth but moderately relaxed natural negative supercoiling, was used to study a transcriptional response of both reporter constructions on minor variations in superhelical density. It is well known that gyrase inhibitors cause accumulation of positively supercoiled topoisomers in the population of plasmid DNAs^25^. Antibiotic concentrations sufficient to compensate highly negative supercoils and generate positively supercoiled topoisomers are rather high. For novobiocin, for instance, 60 min treatment of cells with 80–120 μg/ml of the drug is required. Such treatment greatly disturbs normal growth of bacteria. To decrease this antibiotic-mediated stress, we used low concentrations of nalidixic acid (0.25–2.0 μg/ml), but prolonged time of cultivation (8 h). Such treatment decreased the number of negative supercoiles resulting in the appearance of partly relaxed topoisomers (Fig. 4a) Using the same range of antibiotic concentrations we then estimated changes in the transcriptional efficiency of two reporter genes expressed from either *fep*A/*fes* or *app*Y promoter regions. An asymmetric response to nalidixin was registered in both cases (Fig. 4b). It was detected as a gradual decrease in the ratio of emission intensities I_EGFP_ and I_mCherry_. After prolonged cultivation the effect became even naked eye-visible (Fig. 4b, image below the plot).

For divergent promoters of *app*Y-associated “island” the same relative response to nalidixin was reproduced in the native genomic environment (Fig. 4c). cDNA samples for this experiment were obtained with primers 5 and 1 for sense and antisense RNA-products, respectively (Fig. 3b). The total RNA of bacterial cells grown with (gray bars) or without (dark bars) of 1.5 μg/ml of nalidixin was used as a template. Transcription in sense direction (primers 5 and 4, Fig. 3b) showed no essential drug-dependence, while antisense RNA synthesis (primers 1 and 3) was substantially activated. Relief of the negative supercoiling of the chromosome was confirmed by the expected decrease in the expression level of *fis* mRNA, which is an acknowledged “sensor” of topological changes^26^ (Fig. 4c). Based on this data one can assume that the natural negative superhelicity differentiates activity of divergent promoters supporting that one that can easily perceive topological advantages. Local DNA structural properties in the vicinity of divergently faced promoters, for instance stably curved segments originated from A(T)-tracts^27^ may also contribute to the fine functional adjustment. In this case disturbances in the systems maintaining the desired density of supercoils should equalize activity of divergent promoters, as it was demonstrated in Fig. 4b,c for *app*Y-associated promoters. Relative activity of fepA and fes also showed similar drug-dependence on the plasmid (Fig. 4b) which is in line with nalidixin-mediated dedifferentiation in their strengths. However, the same behavior was observed only in four paired qRT-PCR assays, while cumulative data of all experiments including one opposite response masked this trend (Fig. 4c). Some instability in the activity of fepA and fes already discussed^22^ weakened, therefore, nalidixin-mediated effect. Most probably it is conditioned by almost complete overlap of two promoters with comparable initiation propensity. Topological modulations in this case may be easily reversible and therefore less crucial. Promoter-rich “islands”, providing guaranteed interaction with several RNA polymerase molecules, may be more prone to remodel their own architechture and to stabilize structural alterations introduced by external force, including changes in overall topology.

## Discussion

**Figure 5:**
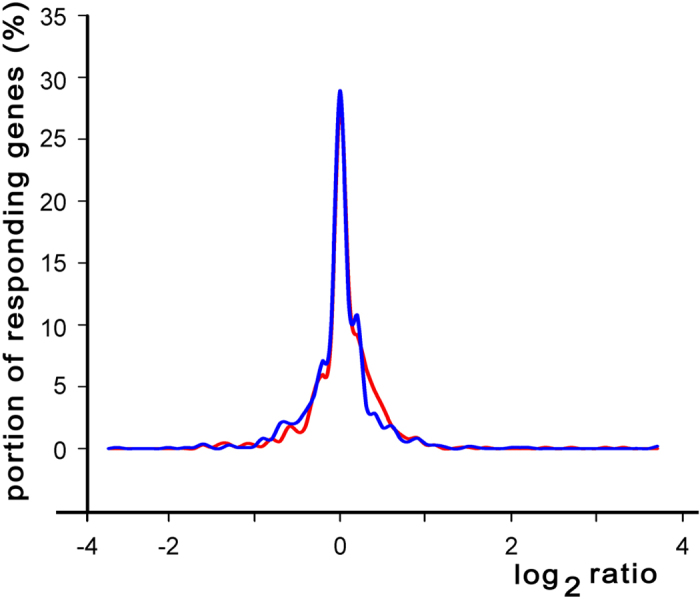
Distribution of genes in respect to changes in expression efficiency caused by 20 min treatment of *E.coli* cells with 1 μg/ml of norfloxacin. The data were obtained by Jeong *et al.*^28^ and are available in GEO database (http://www.ncbi.nlm.nih.gov) (GSE 4408). Genes were subdivided into two groups: the first one was composed of 1020 divergent genes (red line) and the second one included 1082 collinear or convergent genes (blue line). Both sets were prepared using annotation of *E.coli* K12 MG1655 genome available in RedulonDB (http://regulondb.ccg.unam.mx/)^8^.

The involvement of topology-mediated regulation in the control of expression of divergently transcribed genes was inferred from the data of global transcriptional response to the inhibition of DNA gyrase by norfloxacin^28^, subgrouped in this study according to relative positions of corresponding genes (Fig. 5). Thus the distribution curve for log2 ratio of 1020 divergent genes (logarithm of the ratio of hybridization signals obtained for norfloxacin-treated and untreated samples) appeared to be slightly shifted to the right compared to that of 1082 genes with presumed collinear or convergent orientation. Hence, it is likely that divergently transcribed genes are more susceptible for DNA topological modulations at least those with log2 response in the range 0.3–0.7.

Enhanced reactivity of divergent promoters to changes in global superhelical density is consistent with the tenet of supercoiling-dependent transcriptional coupling based in turn on the fact, that positive supercoils are generated ahead of transcribing RNA-polymerase and negative ones – behind the enzyme^7^. The model suggests that mutual interference should be especially significant in the case of promoters located on different DNA strands in “back-to-back” orientation and was indeed thoroughly approved in the case of ilvY and ilvC promoters of *E.coli*^16^. It has been evidently shown that excess of negative supercoiling between two promoters, essential for the activation of ilvC, was accumulated as a consequence of steady-state expression of ilvY. Hence, ilvY and ilvC may by considered as a typical pair of “unequal” partners differing substantially in their capacity to initiate transcription. Each promoter requires certain range of superhelical densities optimal for transcription initiation. This range is shifted to more negative values for the weak promoter ilvC and falls into the physiologically relevant interval for ilvY^29^. The functionality of the strong promoter ilvY, therefore, strengthened a weak promoter ilvC, while the excess of negative supercoiles inhibiting ilvY, still activated ilvC.

In this study quasi-unidirectional response to the treatment by nalidixin was registered only for fepA/fes pair (Fig. 4c). Although variability of qRT-PCR data obtained for these promoters precluded discussing them conclusively, it is worth mentioning that decrease in negative superhelicity activated rather than inhibited transcriptional activity of at least fepA. Such effect is expected if natural superhelicity in the tested genomic region is not optimal for given promoter as it was demonstrated for ilvY/ilvC^29^. Another pair (*bet*I and *bet*T) with unidirectional response to osmotic stress accompanied by considerable changes in DNA topology was described^30^. Mutually consistent reactivity to alterations in superhelical density revealed for *fep*A and *fes* genes is therefore usually observed phenomenon expected from general considerations.

Another genomic region tested in this work demonstrated quite different dependence on gyrase inhibition. Decreased superhelical density expectedly inhibited promoter appY initiating synthesis RNA in sense direction, but strongly and highly reproducible stimulated antisense transcription. In other words, we observed relative changes in the activity of divergent promoters, which assumes different contribution of topological factor to their individual strength or, more likely, some interplay between this topological feature and constraints induced by functioning promoters. Basically, this apparent discrepancy with expected unidirectionality could be explained by the competition between overlapping promoters. The factor of competition for interaction with the same DNA region can overweight advantages given by favorable topological changes and mask expected transcriptional coupling. But in the case of *appY*-associated promoters this explanation is not valuable. Although two divergent promoters operating in this region are partly overlapping (the distance between registered transcription start points is 64 bp), the third one is located far enough (164 bp) to allow independent response to topological changes (see 3D models in Supplementary Fig. 1). Such “anticooperative” relations, when the activity of strong promoter represses moderate divergent promoter located 100 bp apart already have been reported for the *fli*Q of *Caulobacter crescentus* paired with artificial *E.coli* promoter^31^, though the mechanism of this response is not clear.

The plausible clue to explain the anomalous behavior of *app*Y-associated “promoter island” in response to reduced negative superhelicity can be found in the sequence-dependent spatial structure. As a typical representative of “promoter islands” the cloned region has 6 A(T)-tracts that confer it an extremely high propensity to form stable anisotropic bends^27^. They are separated by 15, 24, 40, 14 and 6 base pairs. Being shifted from the exact helical pitch these bends promote the formation of intricate 3D shape of the molecule with a loss of planarity in all 3D models built with different structural parameters (Supplementary Fig. 1). Imposing local supertwisting^18^ these consecutive stable bends may be considered as a primary factor contributing to the quenching or amplification of overall nalidixin-induced superhelical stress.

Obviously, several factors simultaneously contribute to the scale and direction of topology-mediated transcriptional response. For divergent promoters they include background superhelical density in the given chromosomal domain, sequence-dependent individual sensitivity of both counterparts to the level of supercoiling and interpromoter distance. Reporter vector constructed and tested in this study allows studying transcriptional coupling in known environment with easily changeable sequence and structural parameters.

## Methods

### Bacterial strains and plasmids

*E.coli* K12 MG1655 was used for promoter activity assays, while *E.coli* strain E.cloni was utilized for primary selection of fluorescent transformants and for amplification of plasmids containing transcriptionally active inserts. pET-28b (“Novagen”) was taken as a parental plasmid for the construction of the reporter vector. pEGFP-N3 (“Clontech”) and pmCherry-C1 (“Clontech”) were used as a source of genes of fluorescent proteins. All plasmids were isolated using QIAprep Spin Miniprep Kit(Qiagen). Oligonucleotides used for the construction of reporter plasmid are listed in Supplementary Table S1. Intermediate products on the way of dual-colour vector construction were designated as pET28-EGFP, pET28-mCherry, pRBS-EGFP, pRBS-mCherry, final product was named pPF1 (plasmid Promoter Finder). PCR-generated DNA fragment of *tfa*X/*app*Y genomic region (from the position −260 up to position −61 in respect to *app*Y ATG codon) and 857 bp long fragment of *fep*A/*fes* regulatory loci (from the position +449 of *fep*A coding sequence up to position +88 inside the *fes* gene) were inserted into the reporter vector pPF1 by blunt ends ligation into EcoRV restriction site. The presence and quality of inserts were checked by direct sequencing.

### Reporter genes expression assays

Measurements of fluorescence were carried out in sonicated extracts of *E.coli* K12 MG1655 cells transformed by pPF1, expressing reporter genes either from *app*Y-associated “promoter island” or from *fep*A/*fes* regulatory region. Cells were grown in 10 ml of LB medium in 45 ml tubes under aeration and either in the presence or in the absence of nalidixic acid. After 8 h of cultivation, 1.5 ml of culture was withdrawn and cells were harvested by brief centrifugation. The pellet was resuspended in 1 ml of 1xPBS (pH 7.4) and subjected to 20 s sonication on ice. After removing the debris by centrifugation, the cell lysate was diluted up to 3 ml with PBS and fluorescence was measured in 3 ml cuvette by Varian Cary Eclipse Fluorescence Spectrophotometer. The wavelengths of excitation and emission were, respectively, 484 and 507 nm for EGFP or 587 and 610 nm for mCherry.

### 3D structural modelling of appY regulatory region

The sequence of 200 bp DNA-fragment (the same one, which was cloned into reporter plasmid) was submitted to DNAtools server “model it” (http://hydra.icgeb.trieste.it/dna/index.php)^32^. The modelling was performed using structural parameters of trinucleotides (Consensus) and dinucleotides (estimated on the basis of NMR or electrophoretic mobility). 3D models obtained in pdb-format were visualized using PyMOL Molecular Graphics System, Version 1.2r3pre, Schrödinger, LLC (https://www.pymol.org).

### Reverse transcription and quantitative PCR (qRT-PCR)

Transcription start sites mapping in *tfa*X/*app*Y genomic region subcloned in pPF1 was carried out using standard procedure of primer extension. RNA was isolated by extraction with hot phenol. 10 μg of total RNA and 2 pmol of ^32^P- labeled oligonucleotide primer (RFP3 or EGFP3, Supplementary Table S1) were taken for the reaction of reverse transcription with RevertAid Premium Reverse Transcriptase (ThermoScientific). The reaction was performed at 57 °C in conditions specified by manufacturer. cDNA was precipitated by 10-fold volume of N-butanol. Reaction products were separated in 8% PAAG, 8M urea and radioautographed. For real-time PCR analysis cells of *E.coli* K12 MG1655 were harvested after 8 h of incubation with 1.5 μg/ml of nalidixic acid or without it (control sample). The conditions of growth were identical to that ones used for fluorescence measurements. The total RNA was isolated as indicated above, dissolved in 50 μl of 1x DNAse buffer (NEB) prepared on DEPC-water and treated by 4U (2U/μl) RNAse-free DNAse (NEB) for 1 h at 37 °C. RNA concentration was measured spectrophotometrically and confirmed by electrophoresis in 5% PAAG in the presence of 8M urea. Two micrograms of DNA-free RNA were used for reverse transcription with 1 pmol of gene-specific primers. For qRT-PCR the reaction of reverse transcription was performed at 42 °C using RevertAid Reverse Transcriptase (ThermoScientific). Primers used for reverse transcription and qRT-PCR are listed in the Supplementary Table S1. qRT-PCR was performed using DNA Technology termocycler (Russian Federation) and kit qPCRmix-HS SYBR (Evrogen, Russian Federation). Data processing was realized by DNA Technology software. Evaluation of RNA expression levels (E) was done according to ΔCt method (E = 2^−ΔC(t)^). The difference between the threshold values measured for the gene of interest and that of housekeeping reference gene (*rpo*B), ΔCt, was calculated for any given biological replicate. Three technical replicates were done in every biological replicate. Data are presented as a percentage of the expression level of reference gene.

## Additional Information

**How to cite this article**: Masulis, I. S. *et al.* Visualizing the activity of *Escherichia coli* divergent promoters and probing their dependence on superhelical density using dual-colour fluorescent reporter vector. *Sci. Rep.* **5**, 11449; doi: 10.1038/srep11449 (2015).

## Supplementary Material

Supplementary Information
