# Supplementary material for: Visualizing the activity of Escherichia coli divergent promoters and probing their dependence on superhelical density using dual-colour fluorescent reporter vector
Source: Sci Rep. 2015 Jun 17;5:11449. doi: 10.1038/srep11449 (PMC4469952; doi:10.1038/srep11449)

Visualizing the activity of *Escherichia coli* divergent promoters and probing their dependence on superhelical density using dual-colour fluorescent reporter vector

Irina S. Masulis, Zaira Sh. Babaeva, Sergey V. Chernyshov and Olga N. Ozoline

Supplementary Table S1
Oligonucleotides used in this study

Oligonucleotides used for construction of reporter plasmid<br>RBS site is marked in yellow, stop codons – by cyan	
**GFP-NdeI**	5′- TTTTCATATGGTGAGCAAGGGCGAGG -3′
**GFP-XhoI**	5′- TTTCTCGAGTTACTTGTACAGCTCGTCCATGC -3′
**BglII-stops-RBS**	5′- GATCTTAACTAATTAAGTAGCTAGAGTTCTATCAAGAGGTAGC-3′
**NdeI-RBS-stops**	5′- TAGCTACCTCTTGATAGAACTCTAGCTACTTAATTAGTTAA -3′
**stops-BglII**	5′- TCGAGATCTTAACTAATTAAGTAGCTAGAGTTC-3′
**T7term-rev**	5′- CAGCAAAAAACCCCTCAAGACC-3′
**MCS-forw**	5′- GATCTAGAGTCGACAAGCTTGATATCAGAATTCA-3′
**MCS-rev**	5′- GATCTGAATTCTGATATCAAGCTTGTCGACTCTA-3′
Primers used for PCR amplification of *E.coli* genomic regions studied	
**fepA_ forw (1)**	5′- GGACCACGCAGAACTTCAAT-3′
**fes_ forw (4)**	5′- CACCAGTGATGTAGACCCAT-3′
**appY_forw (1)**	5′- GCAAGAGGTTTCAGGTGCGTTGTAGTGAG -3′
**appY_rev (2)**	5′ - CACAATCATAATCAGGATGATGTGCAT -3′
Primers used for reverse transcription assay from culture transformed by appY-pPF1 vector	
**RFP**	5′ - AGCGCATGAACTCCTTGATG -3′
**GFP**	5′ - CGTCCAGCTCGACCAGGATG -3′
Primers used for quantitative RT-PCR	
**fepA_forw (1)**	5′- GGACCACGCAGAACTTCAAT -3′
**fepA_rev (2)**	5′- CGTGGGAATAACCGACAGAT -3′
**fes_forw (4)**	5′- CACCAGTGATGTAGACCCAT -3′
**fes_rev (3)**	5′- GAGAGCTGGTGGCAGTCGAAA -3′
**appY_forw (1)**	5′- GCAAGAGGTTTCAGGTGCGTTGTAGTGAG -3′
**appY_anti_PCR (3)**	5′- CTAACTTATTTCAAAGTTACA-3′
**appY_dir_RT (5)**	5′- CTTAGTTTAGAGGGGCAT-3′
**appY_dir_PCR (4)**	5′- TGCTCCGTAGTTTTCATCTGT-3′
**rpoB_RT**	5′ - CCAAGGCGGTAGCTGACGTATT-3′
**rpoB_PCR**	5′- CACAAGTTCTGGATGTACCTTA - 3′
**fis_PCR**	5′- CCAAAAACCCCTGCGTGACTC- 3′
**fis_RT**	5′- TGCCCATCATCAGCGCAGCA- 3′

Supplementary Fig. S1. 3D structural models of 200 bp DNA-fragment from the *appY* regulatory region build and visualized as described in "Methods" using structural parameters of trinucleotides or dinucleotides as indicated. Atoms in T-tracts are shown by blue spheres, nucleotides in transcription start points are in red. Bent arrows show direction of divergent transcription.

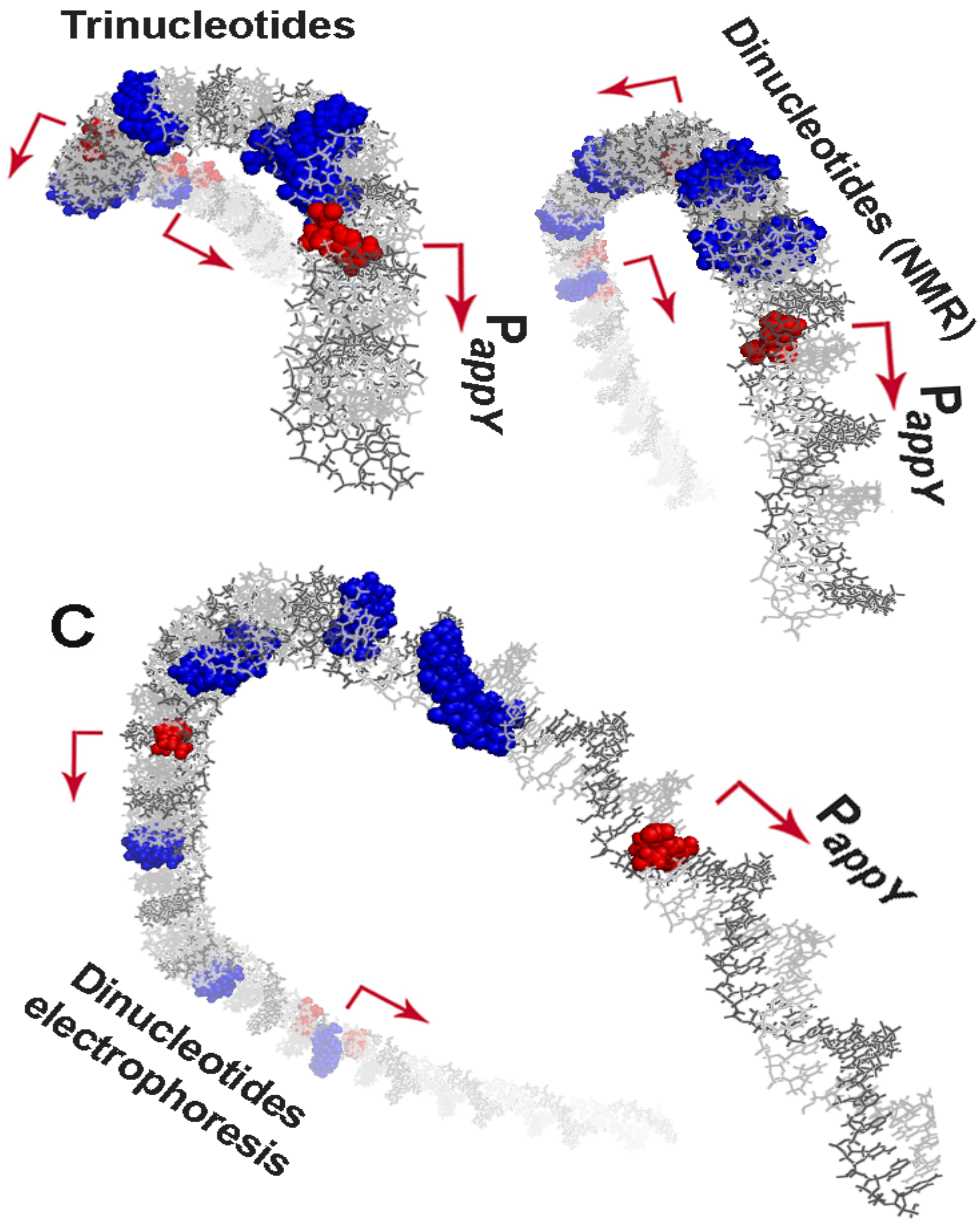

Supplement: Supplementary Information [file srep11449-s1.pdf]
